# Dimensionality Reduction Hybrid U-Net for Brain Extraction in Magnetic Resonance Imaging

**DOI:** 10.3390/brainsci13111549

**Published:** 2023-11-04

**Authors:** Wentao Du, Kuiying Yin, Jingping Shi

**Affiliations:** 1 Nanjing Research Institute of Electronic Technology, Nanjing 210019, China; duwentao_xd@163.com; 2 Department of Neurology, The Affiliated Brain Hospital of Nanjing Medical University, Nanjing 210029, China; profshijp@163.com

**Keywords:** brain extraction, whole-brain segmentation, magnetic resonance imaging (MRI), semantic segmentation, U-Net

## Abstract

In various applications, such as disease diagnosis, surgical navigation, human brain atlas analysis, and other neuroimage processing scenarios, brain extraction is typically regarded as the initial stage in MRI image processing. Whole-brain semantic segmentation algorithms, such as U-Net, have demonstrated the ability to achieve relatively satisfactory results even with a limited number of training samples. In order to enhance the precision of brain semantic segmentation, various frameworks have been developed, including 3D U-Net, slice U-Net, and auto-context U-Net. However, the processing methods employed in these models are relatively complex when applied to 3D data models. In this article, we aim to reduce the complexity of the model while maintaining appropriate performance. As an initial step to enhance segmentation accuracy, the preprocessing extraction of full-scale information from magnetic resonance images is performed with a cluster tool. Subsequently, three multi-input hybrid U-Net model frameworks are tested and compared. Finally, we propose utilizing a fusion of two-dimensional segmentation outcomes from different planes to attain improved results. The performance of the proposed framework was tested using publicly accessible benchmark datasets, namely LPBA40, in which we obtained Dice overlap coefficients of 98.05%. Improvement was achieved via our algorithm against several previous studies.

## 1. Introduction

Over the past few decades, numerous algorithms have been developed and consistently enhanced for the purpose of whole-brain segmentation. These algorithms have now become a crucial element in the field of large-scale neuroscience and neural image analysis research. As the utilization of these algorithms experiences a significant surge, there is a corresponding increase in the demand for enhanced precision and dependability. While the study of fully automated brain extraction techniques has been extensively explored, it remains a dynamic field of research. With the advancement of deep learning techniques, the accuracy of semantic segmentation algorithms for brain tissue has shown significant improvement, attracting considerable attention in the field.

Traditional methods or tools for brain extraction primarily employ a combination of image registration, atlas-based techniques, intensity and edge feature information, as well as level set and graph cutting algorithms to generate accurate brain masks in MRI images. Several algorithms and neural image analysis software have been extensively utilized in the field, which include the Brain Extraction Tool (BET) and its associated algorithms [1,2,3,4], 3DSkullStrip from the AFNI toolkit [5], the Hybrid Watershed Algorithm (HWA) provided by Free Surfer [6], and the robust learning-based brain extraction (ROBEX) method [7]. The deformable spherical mesh model in BET is expanded from its initial position at the center of the image, taking into account local intensity values and surface smoothness. 3dSkullStrip is a variant of BET that incorporates external points to inform the delineation of the grid boundaries. HWA employs edge detection techniques and atlas-based deformable surface models to perform segmentation. ROBEX utilizes a triangular grid that is constrained by the shape model in order to align with the probabilistic output generated by a brain boundary classifier based on random forest methodology. The majority of these algorithms heavily depend on the alignment of the query image with the atlas, or on making significant assumptions regarding the geometry, orientation, and image features. When certain geometric assumptions are not met, the accurate identification of features becomes challenging, and image registration may not guarantee convergence to the exact solution. In numerous instances, the accuracy of these tools’ results is often insufficient due to the inclusion of non-brain structures or the incomplete preservation of brain connections in the segmentation outcomes. Hence, the majority of these tools offer users various options and parameters to configure and experiment with, resulting in brain extraction being a semi-automated or supervised task rather than a fully automated one.

In recent years, image segmentation based on neural networks has garnered significant attention from researchers due to the aforementioned limitations of the traditional methods. A 3D brain extraction algorithm was proposed by Clacek et al. [8], which utilizes seven 3D convolutional layers for the purpose of voxel-level image segmentation. Cubes with dimensions of 53 × 53 × 53 were utilized as input to the network, centered around the gray-scale target voxels. Among the comprehensive evaluations and comparisons documented in the literature [8], the performance surpasses that of state-of-the-art brain extraction algorithms in publicly accessible benchmark datasets. SegNet [9] is a deep convolutional neural network that employs encoders and decoders to perform semantic pixel-level segmentation. The model encoder is comprised of 13 convolutional layers from the VGG16 [10] architecture, which are utilized for downsampling and maximum pooling. Moreover, the pooling coordinates serve to mitigate the loss of pixel location information resulting from the presence of multiple pooling layers. Furthermore, the decoder performs up-sampling by utilizing the corresponding maximum pool index values. Finally, the Softmax classifier is utilized to generate the class-output feature map for each pixel. The U-Net model [11] utilizes the concept of symmetry relationships between encoding and decoding layers to predict medical image segmentation. Two convolutional layers are employed in the encoder to achieve downsampling through the use of maximum pooling. The decoder employs the upper convolution operation to increase the resolution and establish connections with the corresponding feature map size of the encoder. The 3D U-Net model has been widely employed in various applications of volumetric CT and MR image segmentation. These applications include the diagnosis of cardiac structures [12], bone structures [13], vertebral column [14], brain tumors [15,16], liver tumors [17], lung nodules [18], nasopharyngeal cancer [19], multi-organ segmentation [20], head and neck organ at-risk assessment [21], and white matter tract segmentation [22]. The utilization of 3D models, however, results in a significant surge in computational demand, thereby necessitating high-performance computer hardware systems. To address this issue, certain U-Net methods propose employing dimension reduction techniques to enhance processing efficiency. Auto-context 2.5D net for brain extraction [23] incorporates the concept of parameter iteration to enhance the accuracy of segmentation and employs a technique of reducing three-dimensional voxels to three slices, thereby effectively reducing the number of deep learning parameters. The performance of auto-context 2.5D net is close to that of 3D processing, but because the convolution kernel used is the superposition of three 2D planes, the training parameters are still significantly higher than those of 2D processing, and the complexity of processing still has room for optimization. 

In addition, there are currently several directions for optimizing the U-Net using existing modules. These include adding the residual modules to the U-Net network [24,25] to enhance its learning ability, incorporating attention blocks [26] into the encoding and decoding process, and utilizing advanced U-Net connection methods [27] to improve information interaction across different levels. Furthermore, some studies [28] suggest that the U-Net cascade method is highly effective, and optimizing the preprocessing and postprocessing are very important. MVU-Net [29] linearly fused the prediction results of three different views, but the difference in pixel segmentation accuracy under different views is not considered. A 3D CNN or U-Net [30,31] segmentation network makes full use of the characteristics of three-dimensional data, but it has the problem of a large amount of calculation, just like the Auto-CNN method mentioned in [23].

In our collaborative research on brain tissue segmentation algorithms with medical institutions, striking a balance between algorithm complexity and efficacy has consistently been a pivotal consideration. The practical application of software necessitates that we take into account training time and real-time segmentation. 

This paper aims to further investigate the optimization of the overall segmentation method for brain tissue, with a specific focus on the dimensionality reduction hybrid U-Net framework. The following aspects will be covered:

(1) Preprocessing is performed by utilizing the self-supervised global information of the original data, which effectively mitigates the limitations of a localized convolution window. Specifically, the extraction of global amplitude information is efficiently achieved through amplitude-based K-means processing, enabling the realization of clustering and noise reduction. 

(2) The concept of data self-mining is employed to utilize gradient images and construct a hybrid U-Net structure with multiple inputs. We constructed three hybrid U-Net networks and evaluated their performance. The results indicate that incorporating a hybrid fusion approach during the decoding process yields the highest efficiency.

(3) In order to address the issue of a high number of learning parameters in 3D voxel segmentation, an analysis is conducted on the limitations of the two-dimensional U-Net approach. We would like to highlight that the slices exhibiting poor segmentation accuracy typically contain a smaller proportion of brain tissue. We have proposed a combination algorithm following the segmentation of 2D slices, which utilizes multi-dimensional slice information to complement each other and achieves a high level of precision in 3D fusion segmentation accuracy.

As described in nnU-Net [28], the U-Net architecture itself is an excellent structure, and the modification of preprocessing, postprocessing, and some details could significantly improve the network’s performance. In this manuscript, we introduce two new approaches in preprocessing and postprocessing: 

Firstly, in the preprocessing stage, we propose using a clustering method instead of the traditional z-score standardization. Test results show that this approach can improve the classification results of U-Net by about 0.9% in Dice against the traditional technique. 

Secondly, in the post-processing stage, we analyze and point out that the insufficient accuracy of two-dimensional U-Net segmentation mostly occurs in slices with less brain tissue, particularly the slices at the edge of the brain tissue. In other words, the disadvantage of 2D segmentation compared with 3D segmentation mainly occurs in slices with less brain tissue. However, for the sagittal plane, cross section, and coronal plane, the edge of the brain in one of the coordinates may be the middle position of the brain in another or two coordinates. In this way, we comprehensively use the segmentation results of different sections for fusion, that is, the weighted superposition of the classification probability value of each pixel, so as to obtain a better three-dimensional segmentation result. In this way, the calculation amount of directly using the three-dimensional U-Net network can be significantly reduced, and the approximate accuracy can be achieved. 

In addition, we conducted comparisons and tests for multi-input U-Net networks. Specifically, we compared three multi-input networks after the gradient map, using the input image form. One network followed the common approach, while the other two networks considered different levels of structural complexity. The conclusion revealed that all nodes exhibited some degree of performance redundancy. Furthermore, we found that training multiple inputs separately and sharing a decoding link during the coding stage proved to be an efficient approach.

The proposed method achieves superior segmentation results through a series of processing steps while reducing computational requirements. It has been successfully implemented in the neuronavigation system and utilized in numerous accurate navigation treatment studies at Nanjing Brain Hospital. Real-time performance and accuracy of segmentation have been validated, demonstrating promising practical prospects.

In the remainder of this paper, we first provide a global view of our brain extraction method in Section 2.1, then give detailed descriptions of innovative aspects in three separate subsections, and finally verify the performance of the model through experiments on a brain MRI dataset. 

## 2. Materials and Methods

### 2.1. Network Architecture 

**Figure 1:**
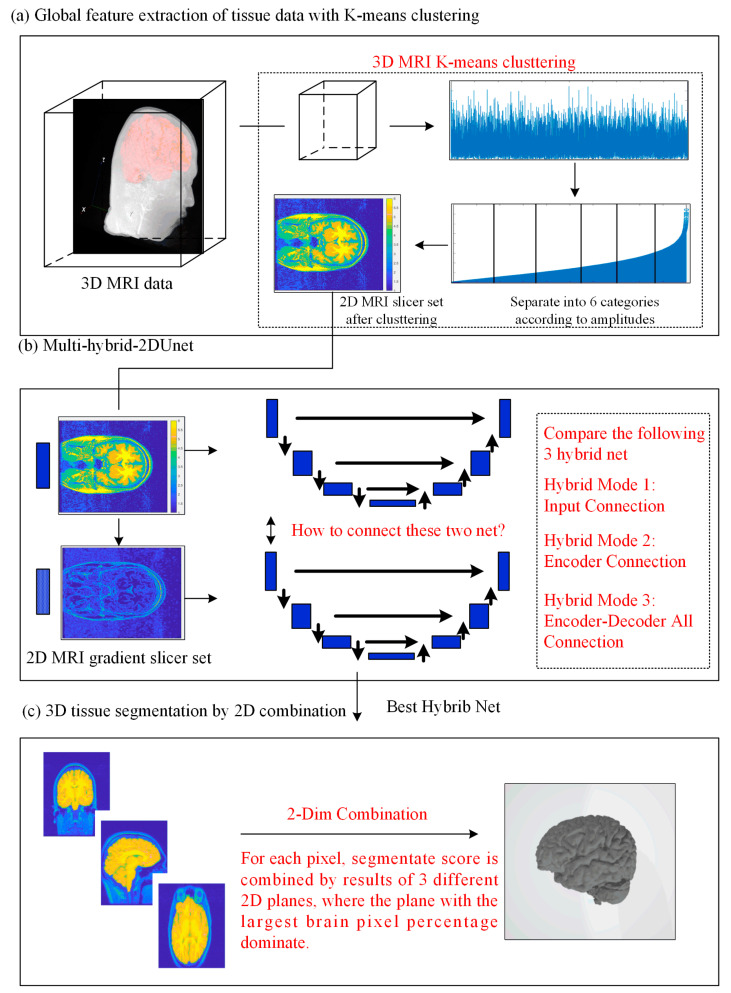
Schematic diagram of the proposed method.

As it is widely acknowledged, U-Net incorporates a framework consisting of an encoder and decoder that relies on convolutional kernels. Therefore, the segmentation outcome would be significantly impacted by the size of the kernel. Restricted by the computational burden and the need for local information extraction, the kernel size is typically not significantly large. The dilated convolution algorithm has been proposed as a means to address this contradiction. However, it should be noted that this approach may result in the loss of local information, as not all points within the window are handled. Additionally, it is important to consider that the size of the window cannot be unlimitedly increased. In this study, we examine the utilization of clustering pre-processing techniques to gather comprehensive intensity data from MRI images. Based on the analysis of pixel intensity across the entire graph, the implementation of clustering pre-processing techniques has the potential to mitigate the limitations associated with conventional kernel methods. The classical K-means clustering algorithm will be employed in this study, as depicted in Figure 1a. The initial 3D voxel tissue will be partitioned into six distinct categories based on the conventional MRI processing software, SPM12 [32].

On the other hand, researchers have endeavored to enhance the efficacy of semantic segmentation through various approaches, ranging from the framework and layers to the intricate calculation methodology. For instance, the loss function is enhanced by incorporating an additional penalty based on the logarithm of the edge of the mask. This modification has the potential to enhance the performance of segmentation in edge areas. Nevertheless, the experimental findings indicate that determining the scales of weighted values is a challenging task, as they are highly sensitive to the data. It is also advisable to concurrently train the edge information data with the original image data. Currently, in the context of U-Net multi-input training, it is common practice to superimpose multiple input data solely at the input layer. This results in the formation of a matrix of *K* × *N* × *M* prior to training. This processing appears to have limitations in effectively extracting information from multi-input data. As depicted in Figure 1b, this study aims to compare the conventional multi-input method with two innovative networks. The first network trains two sets of data separately, each with its own encoder–decoder framework. In this approach, the encoder layers and decoder layers between two inputs are fully connected. The alternative approach involves training two input datasets using two distinct encoder processes, while simultaneously utilizing a single decoder process.

In addition to other factors, enhancing the performance of 3D networks can be achieved through straightforward dimensional extensions. However, it should be noted that 3D networks generally require more computational resources, particularly in terms of memory. Even when utilizing the purported 2.5D processing architecture [23], it is evident that the average runtime is significantly longer compared to the traditional 2D approach. According to the findings of the test results, the utilization of 3D information has been shown to significantly enhance brain edge classification. In our study, it was observed that the 2DU-Net classification model did not perform effectively at the boundary slices of the entire brain. On the basis of the aforementioned, we propose a processing structure that combines different 2D planes, as illustrated in Figure 1c. The 2D-U-Net method is implemented on the axial, coronal, and sagittal planes individually. Subsequently, the segmentation results obtained from each plane are combined using a weighted addition approach, which takes into account the proportion of brain tissue in each slice. Only two-dimensional U-Net processing and low-computation fusion processing are implemented in our algorithm, rendering it a dependable and efficient post-classification processing technique.

Based on the above framework, in the following three sections, we will make a detailed analysis and introduction to cluster preprocessing, multi-input hybrid net, and dimensional reduction reconstruction, which are the most innovative aspects of this study.

### 2.2. Full Image Information Mining with a K-Means Cluster Preprocessing

The convolutional kernel serves as the fundamental computational unit in contemporary deep learning models. The determination of kernel size remains an issue that requires resolution. The local convolution kernel makes it challenging to capture the overall information of the entire image. Dilated convolution is a valuable technique for explicitly modifying the field-of-view of filters and controlling the resolution of feature responses. However, it still has limitations, as it may result in the loss of certain information. In contrast, the traditional K-means algorithm may offer several advantages when applied to MRI images:

(1) Reducing the impact of noise interference. Clustering can be employed as a technique to effectively group low-amplitude noise, thereby mitigating its influence on the outcomes of classification tasks. 

(2) Minimization of amplitude variation within each category. Amplitude fluctuations in pixels within the same tissue are an inevitable occurrence, and the utilization of clustering techniques can effectively mitigate this amplitude variation, thereby enhancing the accuracy of tissue classification.

In fact, numerous conventional techniques for brain tissue segmentation rely on the utilization of clustering algorithms. The CAT module of SPM employs an iterative approach to process brain tissue by means of clustering and registration, with the aim of achieving tissue classification. Hence, in this research, the utilization of K-means clustering is employed as the primary preprocessing technique for the classification of MRI images. The brain tissue is divided into six distinct categories separately, as the SPM [32] did.

### 2.3. Hybrid-U-Net Framework

Currently, in the field of medical image segmentation, several researchers employ a combination of CT, MRI, and other imaging modalities for hybrid training processing. However, in many instances, acquiring diverse format data from testers poses a significant challenge. In the present study, our objective was to utilize a two-dimensional gradient map of the MRI image as supplementary data for hybrid training purposes.

**Figure 2:**
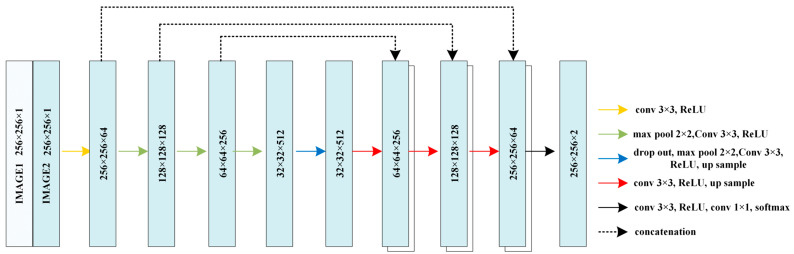
Traditional hybrid input-layer net.

Moreover, in the conventional multi-input hybrid network, it is common practice to concatenate two input images at the input layer. This implies that two images are combined into a single input dataset for subsequent training, as illustrated in Figure 2. It appears that the traditional multi-input network is incapable of fully leveraging the distinct information present in both images. In this paper, we examine two distinct hybrid U-Net frameworks that incorporate the fusion of two input datasets at specific layers during the encoding and decoding processes.

**Figure 3:**
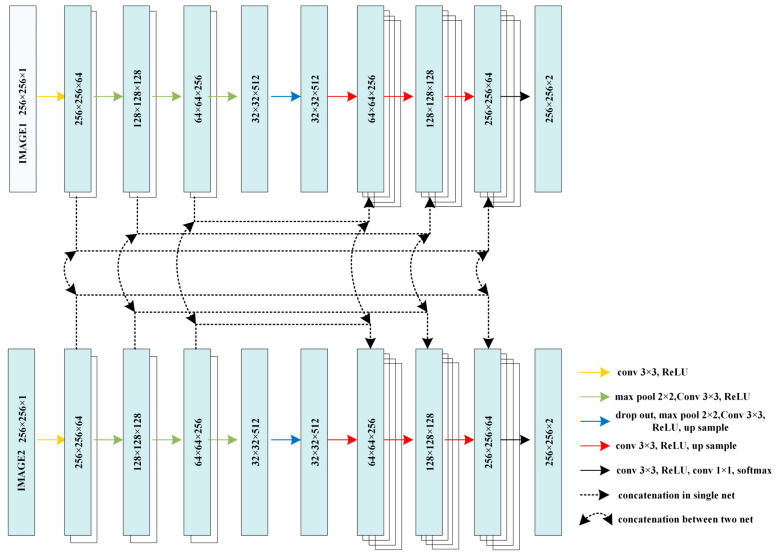
Full connect hybrid U-Net.

As depicted in Figure 2, the U-Net architecture relies on the encoder–decoder procedure as its central framework. The DepthConcatenation, which occurs between the encoding and decoding layers, can be interpreted as a branch. With the inclusion of multiple image input layers, the hybrid U-Net model can have several potential network architectures. Firstly, we propose a fully connected hybrid U-Net architecture. This architecture involves training two complete U-Net models, one for the input image and another for the corresponding gradient image. At each step in the encoder–decoder procedure, the layers between the two models are concatenated, as shown in Figure 3. DepthConcatenation layers are incorporated to establish a connection between the aforementioned networks. Each layer of the DepthConcatenation process combines four images from both networks, resulting in a fused representation.

**Figure 4:**
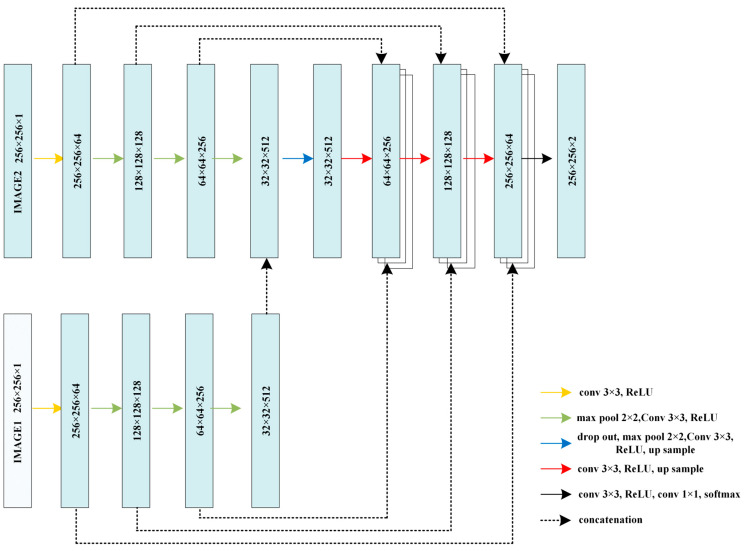
Decoder connection with two separate encoder net.

The aforementioned networks appear to be intricate and may possess certain shortcomings. First, the fully connected network’s skeletons were trained independently on two input images. Secondly, this fully connected network may exhibit a significant level of redundancy. Next, we examine an alternative framework that divides the training process of two input images solely in the encoder section, while merging the decoder section of the two networks, as illustrated in Figure 4. In the subsequent experiments, we will compare the two proposed networks with the traditional input superposition network.

### 2.4. Dimensionality Reduction U-Net for 3D MRI Data

The accuracy of current semantic segmentation in two-dimensional (2D) processing is typically lower compared to the use of three-dimensional (3D) processing. This is because 3D processing has the advantage of utilizing information from adjacent slices and integrating a larger number of pixels, resulting in more precise judgments. Nevertheless, as a result of the substantial expansion of 3D processing parameters, there is a notable increase in both the duration of the training process and the memory resources needed for processing. In fact, a significant body of test results suggests that the benefits of 3D segmentation processing primarily lie in the boundary position, specifically in regions with a lower proportion of brain tissue. The performance of 2D processing in this area is hindered by the absence of auxiliary adjacent slice pixels.

**Figure 5:**
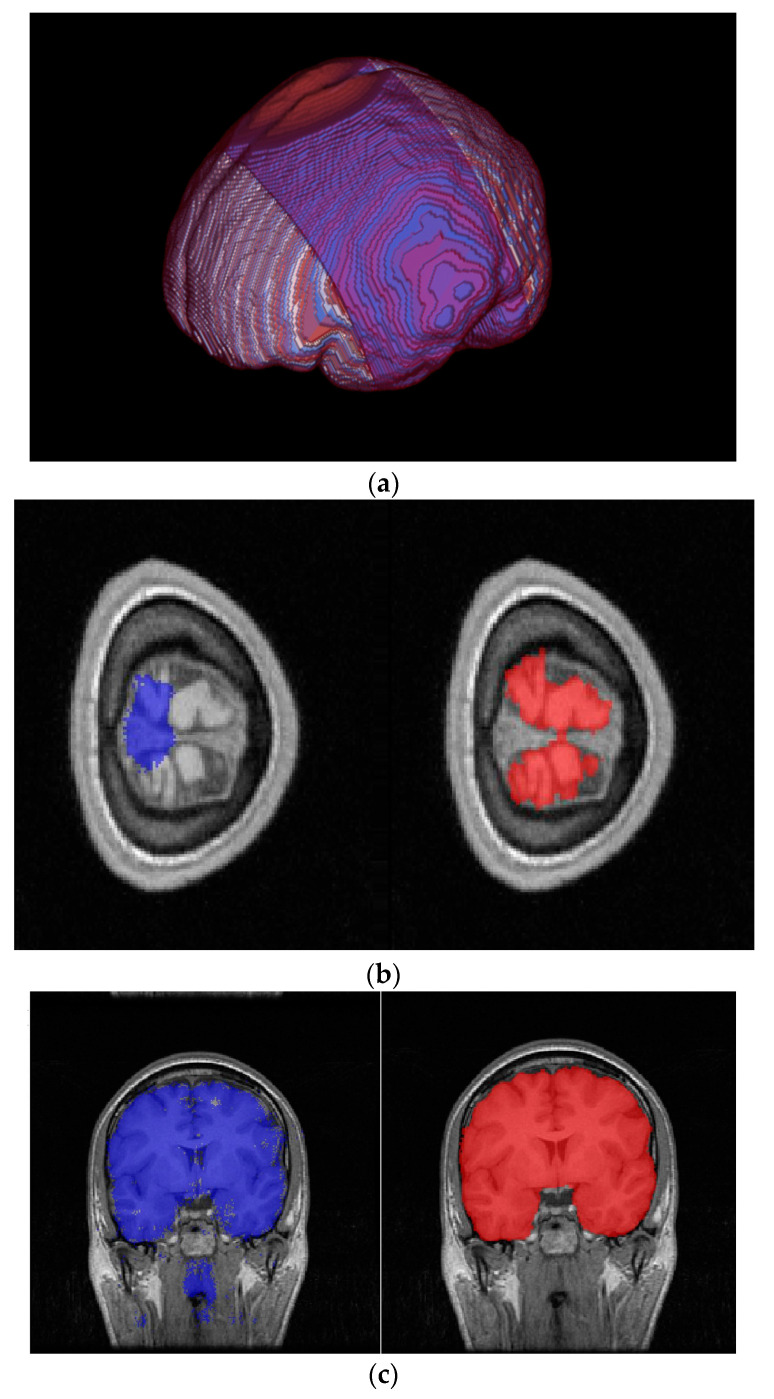
Comparison of two-dimensional U-Net effects of marginal region voxels under different profiles. (**a**) The position of the upper edge region voxel (in red) of the transverse section in the two-dimensional coronal section (in blue). (**b**) Poorly segmented two-dimensional U-net results for edge transverse plane images; the left blue mask shows the poor predicted result with U-Net, and the right red mask shows the label. (**c**) Two-dimensional U-Net segmentation results in the coronal plane; the left blue mask shows the predicted result with U-Net, and the right red mask shows the label.

In light of this phenomenon, we propose a method to enhance the segmentation accuracy of 3D MRI data. This involves decomposing the entire dataset into two or three mutually vertical 2D slice datasets. Each of these sets is then separately trained, and the segmentation results are subsequently fused to improve accuracy. In reality, when examining smaller sections of brain tissue in one plane, it is possible for the tissue to appear more prominent in the other vertical plane. Therefore, combining segmentation results from different planes can enhance the accuracy of 3D classification. As depicted in Figure 5a, central slices in the coronal plane exhibit a greater proportion of brain tissue, as indicated by the blue area. On the contrary, slices located at the upper edge of the transverse plane (red area) exhibit a comparatively lower proportion of brain tissue pixels in the corresponding slices. According to the results of semantic segmentation on a 2D dataset in the transverse plane, it can be observed that the accuracy of semantic segmentation decreases in the edge slices, as depicted in Figure 5b. This decrease in accuracy is identified as the primary factor contributing to the insufficient accuracy of the 2D segmentation. On the contrary, this particular region is part of a larger proportion of brain tissue in the coronal plane slices, leading to improved segmentation performance when using the coronal 2D dataset, as depicted in Figure 5c. Therefore, this characteristic can be utilized to integrate the outcomes of multiple 2D plane segmentations in order to enhance the overall segmentation performance.

**Figure 6:**
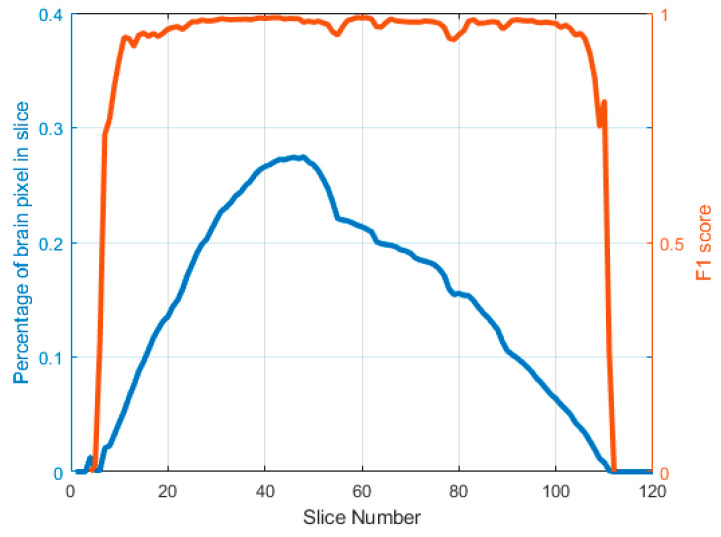
A comparative analysis of prediction accuracy and brain tissue proportion across various serial number sections.

Figure 6 illustrates the relationship between the segmentation accuracy of 2D slices and the proportion of brain tissue pixels in the sagittal plane. The horizontal axis in this figure represents the sequential numbering of 2D slices, ranging from 1 to 120. The blue curve represents the proportion of brain tissue pixels in each specific slice, while the red curve represents the F1 score of U-Net segmentation. It is evident that the accuracy of semantic segmentation significantly increases as the proportion of brain tissue in the entire slice increases. On the contrary, the accuracy of semantic segmentation decreases when the number of brain pixels in one slice is relatively low. This is typically observed in positions with large or small slice serial numbers, which are located near the edge of brain tissue. Hence, the multi-plane fusion architecture proposed in our previous study has the potential to enhance the accuracy of 3D segmentation.

Through the aforementioned analysis, it has been confirmed that the primary factor contributing to the decline in 2D semantic segmentation performance is the reduced proportion of brain tissue at the periphery of the brain. Based on this assumption, the results of pixel segmentation in various sections are integrated and computed using a 2D approach. The softmax classification results are utilized as fundamental parameters, and the proportion of brain tissue in the image is assigned a weight to generate new 3D data representing probability values. The calculation method is presented as follows:

(1) $$P_{c}=w_{1}\cdot P_{1}+w_{2}\cdot P_{2}+w_{3}\cdot P_{3},$$

The symbol $P_{1},{\;P}_{2}\;,{\;P}_{3}$ denotes the semantic segmentation prediction results obtained from the sagittal, transverse, and coronal planes. The symbol $w_{1},{\;w}_{2}\;,{\;w}_{3}$ indicates the weighted values, which are calculated based on the normalized proportion of brain tissue in each section. The main prediction value is attributed to the section with a relatively higher proportion of brain tissue for a specific pixel.

## 3. Results

### 3.1. Datasets

The algorithm under consideration was assessed using benchmark datasets obtained from the LONI Probabilistic Brain Atlas Project (LPBA40, https://www.loni.usc.edu/research/atlases, accessed on 1 December 2022) [33]. This dataset comprises 40 T1-weighted MRI scans of individuals without any known health conditions. The spatial resolution of the scans is 0.86 × 1.5 × 0.86 mm^3^. We employed a five-fold cross-validation approach in all of our experiments. Cross-validation is used to protect a model from overfitting, especially if the amount of data available is limited. In our test, there will be five subsets with equal sizes. In each iteration, the model is trained on one specific subset and validated on the others.

**Figure 7:**
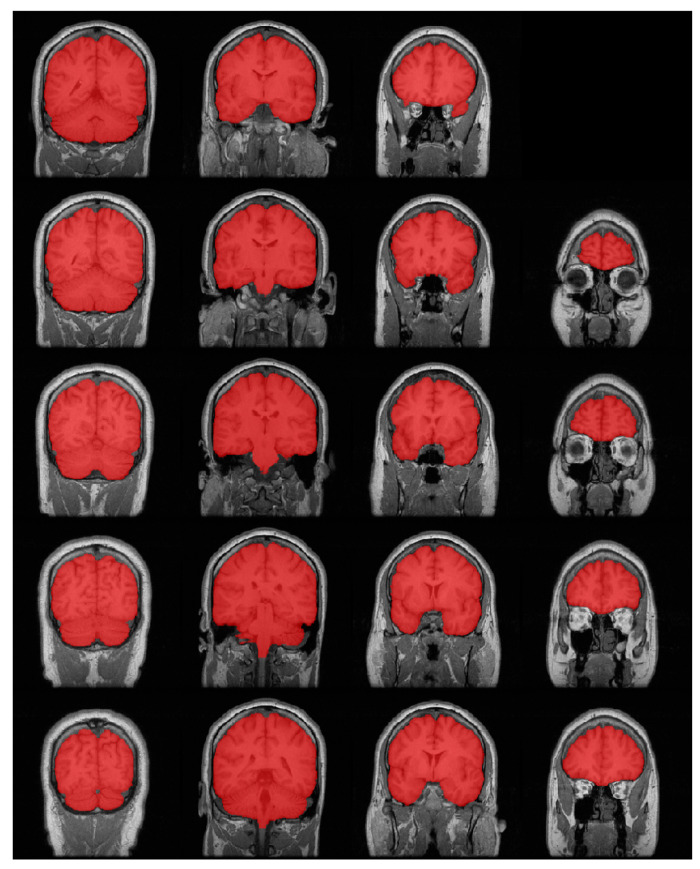
LPBA40 dataset; ground truth mask overlaid on MRI data.

The evaluation of the output from all algorithms was conducted by comparing it to the ground truth. The ground truth was obtained manually prior to this study and was available for the benchmark datasets. Figure 7 shows a set of raw slices with benchmark labels, and we can see that there is a good match between the brain and label. This dataset has been generally used for deep learning brain extraction algorithms [24,26,34], which makes it suitable for testing the algorithm in this manuscript. According to the segmentation results of previous studies, we can see good performance with UNet with this dataset. In this manuscript, we will continue to test our algorithm with this dataset and compare the results with previous studies.

In this experiment, the ADAM optimizer will be used as the optimizer with a learning rate of 1 × 10^−3^. All the experiments are performed using an NVIDIA Tesla V100 GPU with 32 GB of memory. Models are trained and compared using the Deep Learning Toolbox in Matlab (version 2020a).

### 3.2. Results

To assess the efficacy of the algorithms, the Dice overlap coefficient was employed to compare the predicted brain mask (*P*) with the ground truth mask (*R*) that was manually extracted. The Dice coefficient was calculated using the following formula:

(2) $$D=\frac{2\left|P\cap R\right|}{\left|P\right|+\left|R\right|}=\frac{2TP}{2TP+FN+FP}$$

The terms *TP*, *FP*, and *FN* represent the true-positive, false-positive, and false-negative rates, respectively. The symbol denotes the process of summing over all the elements. We additionally present specificity and sensitivity as metrics for comparing algorithms. Sensitivity represents the ability of brain extraction methods to correctly recognize brain tissue, and specificity represents the ability of brain extraction methods to correctly recognize non-brain tissues, which are calculated using the following formulas:

(3) $$F\mathrm{specificity}=\frac{TN}{TN+FP}$$

(4) $$F\mathrm{sensitivity}=\frac{TP}{TP+FN}$$

Table 1 presents the outcomes of our proposed approach in comparison to several alternative methods across the evaluated datasets. For comparison, the results of the classical BET algorithm as well as the recently proposed Auto-U-Net are given, which are taken from [23]. Our algorithm showed the highest Dice coefficients among all methods, with an increase of about 0.32% over the Auto-U-Net methods in the LPBA40 dataset. The improvement in performance was achieved through the proposed pre-processing hybrid training and 2D combination framework, which is named PHC-U-Net for short in the following part of this manuscript.

The utilization of cluster preprocessing in U-Net demonstrates a notable enhancement of 0.9% compared to the conventional approach. This finding provides evidence that reducing the pixel amplitude distribution through cluster preprocessing significantly improves the effectiveness of U-Net. Subsequently, a comparison was conducted to assess the impact of employing three distinct multi-input networks following preprocessing. Hybrid 1 is the traditional input layer fusion method (Figure 2). And hybrid 2 encodes separately while sharing one decoding network (Figure 4). Then, hybrid 3 trains the two input images separately in the entire encoding and decoding net, connecting these two nets through deep connections (Figure 3).

The test results of the three different hybrid nets show that the second hybrid mode exhibits the best performance. According to our previous analysis, the performance of the first net is insufficient because it only concatenates the images at the input layer. The primary factor contributing to the lack of significant improvement in the performance of the third mode could be attributed to the separate training of the two constructed networks, which may have hindered effective fusion.

Finally, using two-dimensional combination processing can reduce the error in the edge region segmentation, which helps to further improve the segmentation accuracy.

**Figure 8:**
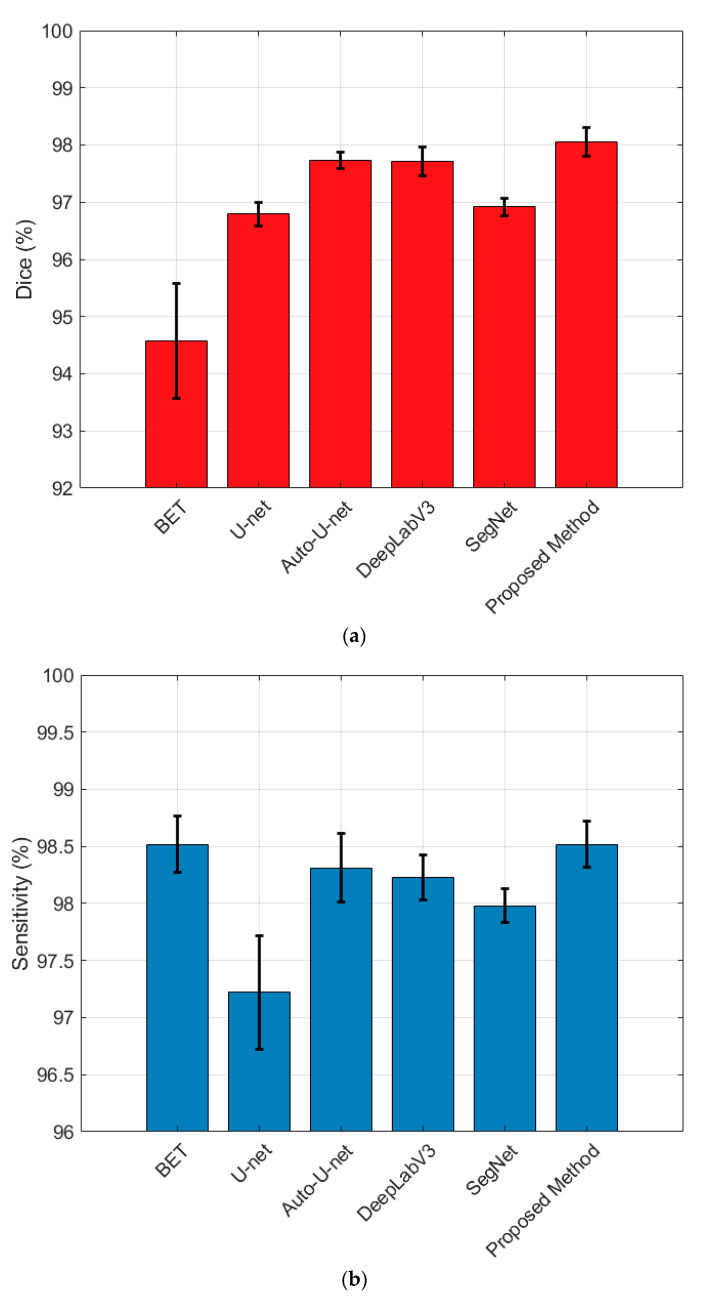

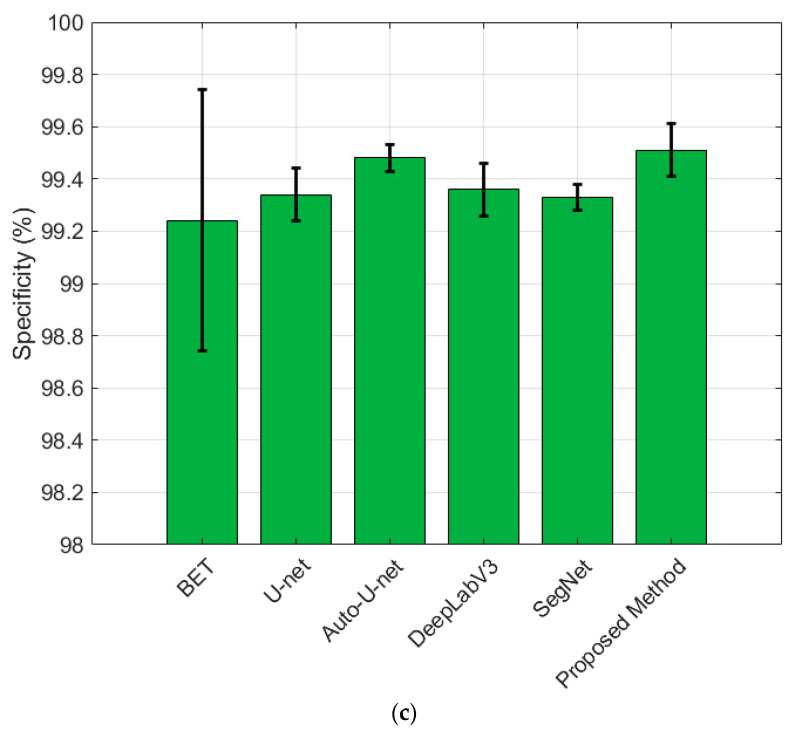
Comparison of evaluation scores of BET, U-Net, Auto-U-Net, and the proposed method: (**a**) Dice; (**b**) sensitivity; (**c**) specificity.

For ease of observation, Figure 8 shows the quantitative comparisons between our methods and other skull-striping algorithms according to the results of Table 1. As we can see, the proposed method resulted in the highest Dice score with the testing datasets and outperformed the traditional U-Net and BET. Similar results to Auto-U-Net can be achieved with the proposed K-means preprocessing, whereas the proposed algorithm has a lower computational burden than Auto-U-Net. Both hybrid processing and two-dimensional combination techniques have been found to enhance performance. Consequently, the PHC-U-Net model yields the highest level of performance.

**Figure 9:**
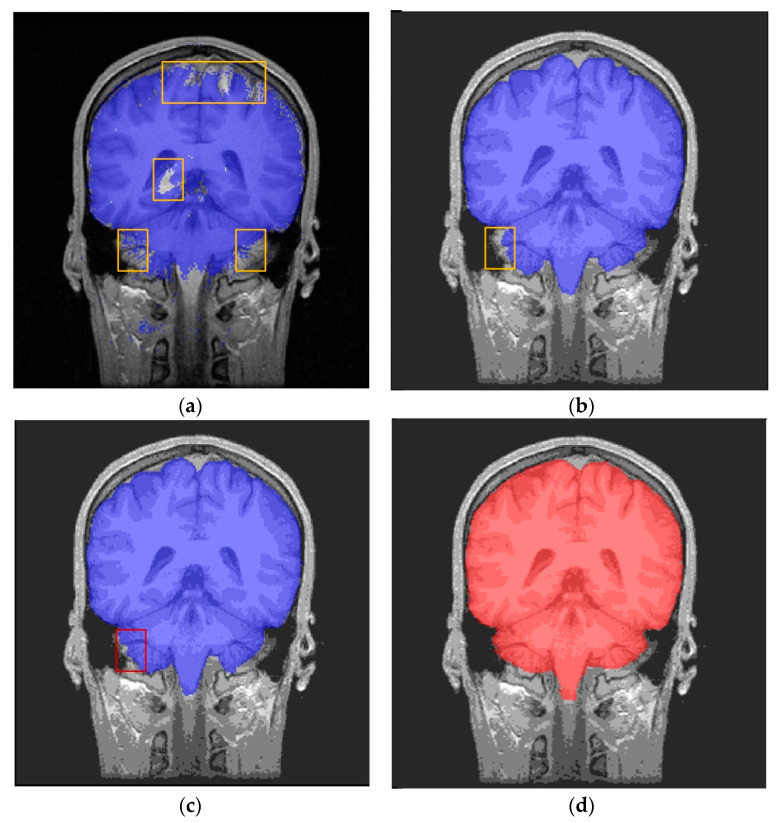
The predicted masks superimposed on MRI data in the coronal plane. These four images show the improvement of the predicted brain mask in different steps of the proposed algorithm. (**a**) The predicted brain mask with the original U-Net, and several deficiencies are marked with boxes in yellow. (**b**) The prediction performance is improved with K-means preprocessing, whereas one can still see the drop-off at the edges of the brain. (**c**) The image demonstrates the enhancement of the edge area through the utilization of a hybrid net, which can be seen within the red box. (**d**) The overlay of the referenced label mask onto the MRI data.

With respect to [35], the gradient map of the pre-processed image was utilized as an additional input image, encompassing the grayscale trends of the pixels. The test results indicate that the utilization of a two-input hybrid net can enhance the amount of information available for training purposes. We additionally conduct tests on various other images, including the edge map, for hybrid training purposes. However, the results show no improvement in performance but a certain decline. The reason for this phenomenon could be attributed to the fact that the information obtained through the convolution kernel is more comprehensive compared to edge detection. Consequently, the conventional edge detection method may result in the loss of certain information. Figure 9 presents a comparison of the predicted outcomes obtained from three different models: U-Net, U-Net with K-means as a preprocessing step, and a two-dimensional hybrid U-Net model. It can be seen that the performance of pre-processed U-Net is significantly better than that of original U-Net, which can effectively reduce the mistaken ‘holes’ in the prediction results. Meanwhile, the performance of the hybrid network can be further enhanced by incorporating gradient images in specific edge regions. In this slice, the performance improvement of the 2D combination is negligible.

**Figure 10:**
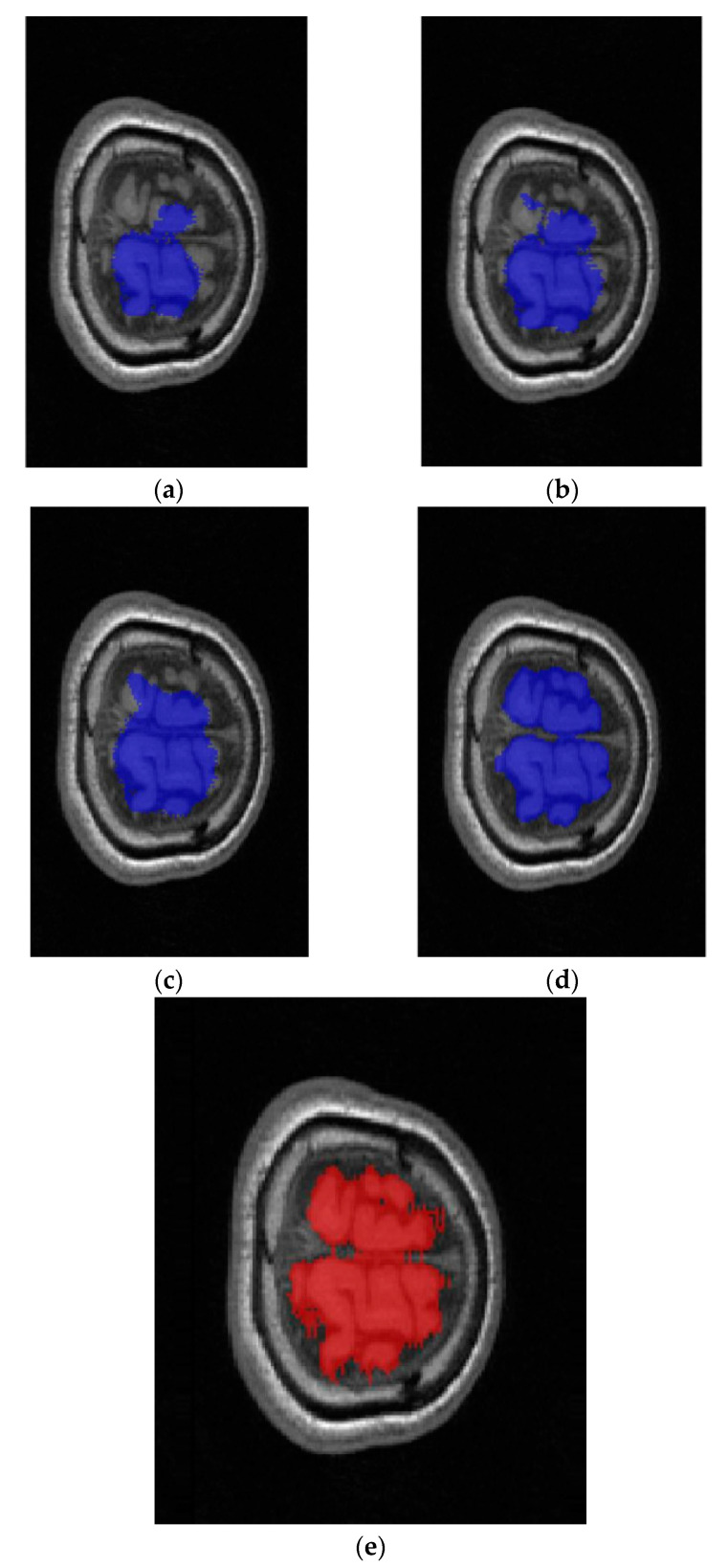
Predicted masks overlaid on MRI data (transverse section). (**a**) The brain mask predicted using the original U-Net model. (**b**) The improvement in prediction performance achieved through K-means preprocessing. (**c**) The enhancement in the edge area achieved by employing a hybrid net. (**d**) The good performance obtained using the PHC-U-Net model. (**e**) The superimposed reference label mask on the MRI data.

To demonstrate the efficacy of the two-dimensional combination algorithm, a comparison is made between the outcomes of U-Net, U-Net with K-means as a preprocessing step, a two-dimensional hybrid U-Net network, and the proposed PHC-U-Net algorithm, as depicted in Figure 10. The prediction accuracy gradually increases with each processing step, and finally, the overall PHC-U-Net framework can greatly alleviate the problem of segmentation ability degradation caused by insufficient pixel units at edge areas.

The proposed method is essentially a 2D U-Net model and yields low computational costs. The model parameter amount of the algorithm in this paper is less different from that of conventional UNet, about 40% of the parameter amount of Auto-U-Net, and about 10% of 3D U-Net. In terms of test time, the length of the processing time is 4.7 s, about 45% of the Auto-U-Net. As to memory usage, under the selected mini-batch size, the occupied memory of our algorithm is also significantly lower than that of 3D U-Net and Auto-U-Net.

## 4. Discussion

Compared with the traditional brain segmentation methods, the UNET-based methods obtain the features of images through training without the need to extract features manually. Meanwhile, UNET methods also do not need image registration, which is almost a necessary process in traditional segmentation methods. For UNET methods, some recent articles have optimized the performance by changing the encode and decode modules or adding some new deep learning frameworks with UNET. In this paper, we try to provide a more basic optimization idea, that is, how to improve the performance of two-dimensional processing through preprocessing, postprocessing methods, and a simple multi-input network to obtain a better three-dimensional segmentation effect. This kind of method is applicable or partially applicable to most UNET-based networks.

Based on the experimental results in Section 3, the dimensionality reduction UNET network that we proposed is superior to several recently proposed deep learning methods and some classical brain extraction technologies. We achieved the highest Dice coefficient by striking a balance between performance and efficiency. This was achieved through the utilization of clustering preprocessing, multi-input hybrid training, and postprocessing dimensionality reduction on the standard dataset.

Although clustering methods have been widely used in traditional image matching and segmentation, we first applied them to deep learning preprocessing, and their performance showed a very significant improvement, reaching about 0.9%. This has very important practical significance; that is, using very simple processing can obtain the approximate effect of complex network structure changes, which also confirms what nnUnet proposed: that preprocessing is an important step in improving the performance of Unet. The amplitude range of MRI data is relatively large, but for brain segmentation, such a fine and complex pixel amplitude distribution is not required. On the contrary, a limited pixel distribution will help the effect of brain segmentation. Other medical images may have similar results, and we will continue to investigate them in subsequent studies.

When considering mixed input, it can be observed that the structure that shares a common decoding part has better performance than the structure that connects all the corresponding layers. This is also a very meaningful conclusion, because with the development of UNET, there are unet+, unet++, and unet3+ network configurations, and more and more nodes are connected together. However, for multi-input networks, there is no evidence to support the conclusion that connecting more nodes will result in better performance. As shown by our test data results, for a multi-input UNET structure, there may be redundancy in the interconnection between the layers within the coding component, and the effect of using the same decoder may be better.

In this paper, we also focus on dimensionality reduction processing, specifically using 2D Unet to approximate the results of 3D processing. This is important for some medical institutions that do not have high-performance computers, and also helps to reduce the complexity of the model, so as to continue to add other modules in subsequent studies. As described in the article [23], UNET does not require a large structural change when extended to a 3D network, but 3D networks usually have higher requirements on computing resources, especially memory. We utilize three sets of two-dimensional networks to simulate the impact of three-dimensional processing, which can effectively decrease the memory requirements during processing.

## 5. Conclusions

In this manuscript, we present a novel framework that incorporates modifications to the hybrid U-Net model for the purpose of brain tissue segmentation. The proposed method incorporates three enhancements in the areas of preprocessing, hybrid framework, and two-dimensional fusion. These improvements effectively reduce the network size while maintaining a satisfactory level of segmentation accuracy. It should be noticed that our proposed clustering preprocessing method and hybrid input framework have the potential to serve as a general processing approach for a wide range of medical image deep learning segmentation methods. The utilization of a two-dimensional segmentation fusion architecture can effectively mitigate the hardware requirements in scenarios involving high-pixel and large-volume data. Additionally, this approach is beneficial for enhancing training speed.

It is possible to deduce the subsequent conclusions briefly from the test results:

(1) The application of clustering for data preprocessing demonstrates a notable enhancement in brain tissue segmentation. The presence of grayscale variations within the same tissue poses a challenge for deep learning classification. After the process of clustering, the grayscale values of the tissue undergo compression, resulting in a narrower range. Consequently, the utilization of U-Net yields improved outcomes, particularly in terms of reducing misclassification areas.

(2) When considering hybrid inputs, it was observed that the architecture that shared a common decoding part of U-Net performed better than the architecture that connected all corresponding layers. In essence, it is possible for redundancy to be present in the interconnections between layers within the encoding component.

(3) The results of 2D section segmentation are integrated to form 3D data by considering the proportion of brain tissue. This combination approach demonstrates comparable performance to the established three-dimensional U-Net method while also substantially reducing computational complexity and hardware demands.

However, for the data on lesions or infants, the effectiveness of the method proposed in this paper needs to be further tested in subsequent studies. In particular, reducing the pixel gray distribution may affect the segmentation effect of this special brain tissue, and may need to be optimized specifically, which will be considered in future studies.

## Figures and Tables

**Table 1:** The mean and standard deviation of the three evaluations of the public dataset LPBA40 with different brain extraction methods.

Method	Dice	Sensitivity	Specificity
BET	94.57 (±0.02)	98.52 (±0.005)	99.24 (±0.01)
U-Net	96.79 (±0.004)	97.22 (±0.01)	99.34 (±0.002)
Auto-U-Net	97.73 (±0.003)	98.31 (±0.006)	99.48 (±0.001)
DeepLabV3+	97.72 (±0.005)	98.23 (±0.004)	99.36 (±0.002)
SegNet	96.92 (±0.003)	97.98 (±0.003)	99.33 (±0.001)
Preprocessing-U-Net	97.69 (±0.005)	98.27 (±0.007)	99.39 (±0.002)
Prepro-Hybrid1-U-Net	97.68 (±0.004)	98.28 (±0.003)	99.38 (±0.003)
Prepro-Hybrid2-U-Net	97.76 (±0.003)	98.33 (±0.004)	99.49 (±0.005)
Prepro-Hybrid3-U-Net	97.72 (±0.002)	98.29 (±0.003)	99.41 (±0.004)
Prepro-Hybrid2-U-Net with 2D combination (PHC-U-Net)	98.05 (±0.005)	98.52 (±0.004)	99.51 (±0.002)

## Data Availability

The proposed algorithm was tested using benchmark datasets obtained from the LONI Probabilistic Brain Atlas Project (LPBA40).
